# Cognitive‐Motor Conflict During Step Initiation: Neural and Biomechanical Insights Into Freezing of Gait in Parkinson's Disease

**DOI:** 10.1111/ejn.70374

**Published:** 2025-12-20

**Authors:** Claudiane Arakaki Fukuchi, Luana dos Santos de Oliveira, Claudia Eunice Neves de Oliveira, Layla Cupertino, João Ricardo Sato, Margarete de Jesus Carvalho, Fabio Augusto Barbieri, Daniel Boari Coelho

**Affiliations:** ^1^ Human Movement Research Laboratory (MOVI‐LAB), Department of Physical Education, School of Sciences São Paulo State University (UNESP) Bauru São Paulo Brazil; ^2^ Center for Mathematics, Computation and Cognition Federal University of ABC São Bernardo do Campo São Paulo Brazil; ^3^ FMABC University Center Santo Andre São Paulo Brazil; ^4^ Biomedical Engineering Federal University of ABC São Bernardo do Campo São Paulo Brazil

**Keywords:** anticipatory postural adjustments, biomechanics, brain activity, fNIRS, motor control, movement disorders

## Abstract

Freezing of gait (FoG) is a disabling motor symptom in Parkinson's disease (PD) characterized by brief episodes of inability to step, often triggered by cognitive‐motor conflict. Step initiation (SI) involves anticipatory postural adjustments (APAs), which are critical for balance and movement preparation. This study aimed to investigate the neural and biomechanical correlates of FoG during SI under congruent and incongruent conditions, focusing on the supplementary motor area (SMA) and dorsolateral prefrontal cortex (DLPFC), regions associated with motor preparation and inhibitory control. Thirty‐three individuals with PD (15 with FoG and 18 without FoG) performed a stepping initiation task under two conditions: congruent (aligned visual cues) and incongruent (conflicting visual cues). APAs were analyzed using biomechanical data, while functional near‐infrared spectroscopy measured oxyhemoglobin (oxy‐Hb) concentrations in the SMA and DLPFC. Two‐way ANOVA assessed differences between groups and conditions. Incongruent conditions resulted in higher APA amplitude, duration, delay, and task error rates than congruent conditions. Individuals with FoG exhibited reduced SMA activation compared to those without FoG, particularly during incongruent tasks. DLPFC activation was greater during incongruent conditions but showed no significant differences between groups. This study highlights the critical role of the SMA in managing cognitive‐motor conflict during SI in PD. Reduced SMA activity and impaired APAs in FoG individuals underscore the need for targeted interventions addressing both cognitive and motor deficits to improve gait and reduce FoG episodes. These findings suggest a dissociation between motor preparation and inhibitory control in individuals with FoG.

## Graphical abstract

(A–D) Illustration of the task execution: (A) preparation phase, (B) correct trial (stepping with the correct leg and one APA), (C) incorrect trial (stepping with the correct leg but two APAs), (D) incorrect trial (stepping with the wrong leg). (E) Representation of the functional near‐infrared spectroscopy (fNIRS) probe layout used in the experiment, highlighting the regions of interest: supplementary motor area (SMA) and bilateral dorsolateral prefrontal cortices (DLPFC). (F) Boxplots of the APA amplitude, APA duration, APA delay, task error and SMA and DLPFC activation for the FoG and nFoG groups during congruent and incongruent conditions. (*p < 0.05: statistically significant differences between conditions, #*p* < 0.05: statistically significant interaction groups and conditions).

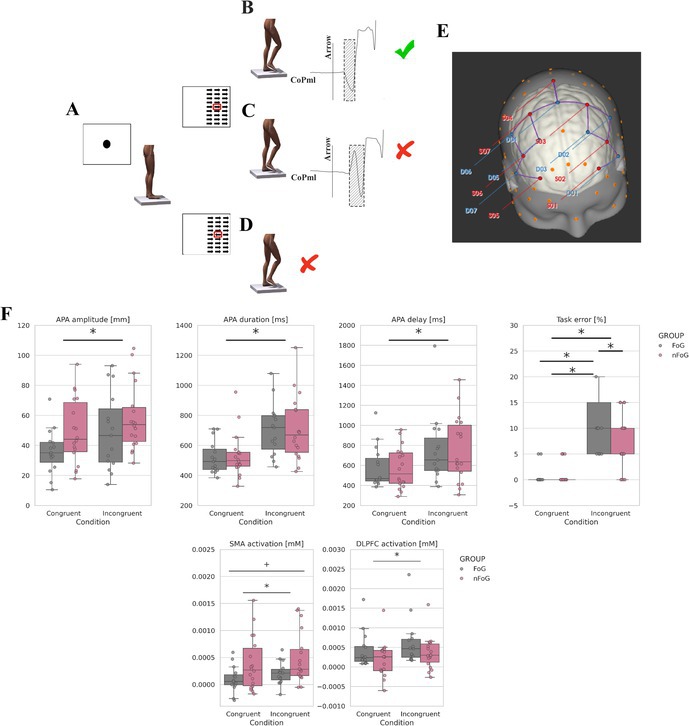

**Abbreviations**
ANOVA: analysis of variance
APA: anticipatory postural adjustment
CoP: center of pressure
DLPFC: dorsolateral prefrontal cortex
fNIRS: functional near‐infrared spectroscopy
FoG: freezing of gait
GLM: general linear model
H&Y: Hoehn & Yahr stage
HbO (oxy‐Hb): oxygenated hemoglobin
HbR (deoxy‐Hb): deoxygenated hemoglobin
ML: mediolateral
MoCA: Montreal Cognitive Assessment
PD: Parkinson's disease
ROI: region of interest
SMA: supplementary motor area
UPDRS‐III: Unified Parkinson's Disease Rating Scale, Part III (motor examination)
*η* ^*2*^
: eta‐squared (effect size)

## 1 Introduction

Step initiation (SI) is a complex motor task that involves transitioning from a stationary standing posture to dynamic balance to initiate forward movement. This process integrates motor and cognitive aspects, including anticipatory postural adjustments (APAs) and movement execution. APA involves subtle preparatory movements that enable the body to maintain balance in preparation for SI. Effective SI requires controlling body balance, as indicated by APAs, such as shifting the center of pressure (CoP) laterally and posteriorly to move the body's center of mass toward the stance leg, thereby allowing the body to move forward in the direction of movement progression (Gélat et al. 2006).

Individuals with Parkinson's disease (PD) frequently experience impaired ability to initiate this transition, resulting in an inability to transition from standing to walking. This impaired ability to transition is a significant contributor to falling risk. A key issue in PD is the dissociation between APA and step onset (Jacobs, Nutt, et al. 2009), where postural preparation and movement initiation become decoupled. Compared to healthy controls, individuals with PD show a noticeable delay in the sequencing between the initiation of APA and the onset of the first step (Mancini et al. 2016), contributing to postural instability and difficulty in executing quick, accurate steps in environmental hazards (Caetano et al. 2019). This disruption is particularly pronounced in individuals with freezing of gait (FoG), who often exhibit inappropriate APAs, particularly during attentional tasks (Smulders et al. 2015).

Cognitive deficits, particularly in executive functions such as inhibitory control, are also common in individuals with PD and FoG, making it challenging to release inhibition (Cohen et al. 2014). In tasks that require conflict resolution and response inhibition, individuals with PD step substantially more slowly and make more errors than healthy age‐matched controls (Caetano et al. 2018). Moreover, when comparing a simpler to a more complex task, APA onset occurred 88 ms earlier, where individuals with PD without FoG initiated APA later than healthy controls (Cohen et al. 2017). Additionally, APA duration was longer in individuals with PD, with those experiencing FoG exhibiting a longer duration than those without FoG, compared to healthy controls (De Oliveira, Silva‐Batista, et al. 2025). These deficits, mainly in the presence of FoG, are linked to disruption in the frontostriatal circuitry, which is important for selecting and inhibiting actions (Mostofsky and Simmonds 2008).

Neuroimaging studies suggest that APAs during SI involve high‐level cognitive processing, particularly in the supplementary motor area (SMA) and dorsolateral prefrontal cortex (DLPFC). While SMA is thought to inhibit prepotent neural commands during SI, preventing the execution of an inappropriate step for the given context (Albares et al. 2014), the DLPFC is involved in signaling APA errors (Watanabe et al. 2016). Although reduced cortical activity in the SMA and DLPFC during complex stepping tasks has been observed in individuals with PD (Pelicioni et al. 2020; De Oliveira, De Oliveira, et al. 2025), these findings were compared only with healthy controls, and FoG episodes were not explicitly accounted for. A prior study from our group directly compared PD with healthy controls during a cognitively demanding SI task. Using fNIRS, De Oliveira, De Oliveira, et al. (2025) examined participants with PD and healthy controls. They identified group differences in APAs and in cortical activation of the SMA and DLPFC under congruent versus incongruent conditions, using age‐matched groups. These findings provide the PD vs. control context for the present study, which targets disease‐intrinsic mechanisms by contrasting PD with and without FoG.

In PD individuals with FoG, the SMA exhibits decreased functional and structural connectivity with the subthalamic nucleus (STN) via the hyper‐direct pathway, which modulates motor response inhibition (Aron and Poldrack 2006). Similarly, reduced connectivity between the DLPFC and the basal ganglia has been observed. These disruptions in frontal network control may contribute to APAs' deficits during SI in PD individuals with FoG. Although FoG affects the function and efficiency of the motor cortex circuitry (Shine et al. 2013) and worsens APA performance (Delval et al. 2014; Beaulne‐Séguin and Nantel 2016; Cohen et al. 2017) during SI in cognitive tasks of inhibitory control, the neural correlates of APA impairments during inhibitory tasks in individuals with FoG remain unclear. Thus, this study investigated SMA and DLPFC activity during cognitive conflictual stepping, i.e., when inhibition is required to correctly select the step to initiate gait, in individuals with PD with FoG and those without FoG (nFoG). We hypothesized that individuals with FoG would exhibit reduced SMA and DLPFC activation during cognitively conflictual stepping tasks and impaired APAs compared to those without FoG.

## 2 Materials and Methods

### 2.1 Participants

Thirty‐three individuals with PD, diagnosed by a neurologist according to UK Brain Bank criteria (Hughes et al. 1992), volunteered for this study. They were divided into two groups: individuals with FoG (FoG, *n* = 15) and individuals without FoG (nFoG, *n* = 18). The New Freezing of Gait Questionnaire (Nieuwboer et al. 2009) was used to determine FoG or nFoG status. Inclusion criteria were age over 60 years old, no orthopedic issues, no cognitive decline (MoCA score > 19), mild or moderate PD (Hoehn & Yahr I, II or III), no other neurological impairment, no deep brain stimulation (DBS), and stable PD medication for at least 3 months. We restricted enrollment to individuals aged ≥ 60 years to minimize age‐related heterogeneity in spatiotemporal gait, APAs, and cortical hemodynamics. Participants with MoCA < 19 were excluded. This conservative threshold was selected to avoid moderate cognitive impairment that could interfere with instructional comprehension, dual‐task performance, and consistent execution of step‐initiation trials; our goal was to assess APA control and cortical responses without confounding from substantial cognitive deficits. Participants with DBS were excluded because stimulation parameters may modulate SMA/frontoparietal networks and gait circuitry, potentially confounding cortical activation and APA outcomes under our protocol. Before data collection, each participant read and signed the consent form approved by the Federal University of ABC's Ethics Committee (CAAE #06090919.4.0000.5594).

### 2.2 Procedures

Testing was conducted in the “on” state, approximately 60 min after dopaminergic medication intake (Araújo‐Silva et al. 2022). This choice reflects typical daily functioning and aligns with common practice in clinical gait studies, enhancing ecological validity and safety. Our prespecified objective was to analyze APA control during SI independent of the immediate occurrence of FoG, rather than to maximize FoG incidence per se. Clinical assessments included the Hoehn & Yahr (H&Y), UPDRS‐III motor score, and the Montreal Cognitive Assessment (MoCA).

### 2.3 Experimental Task

**FIGURE 1:**
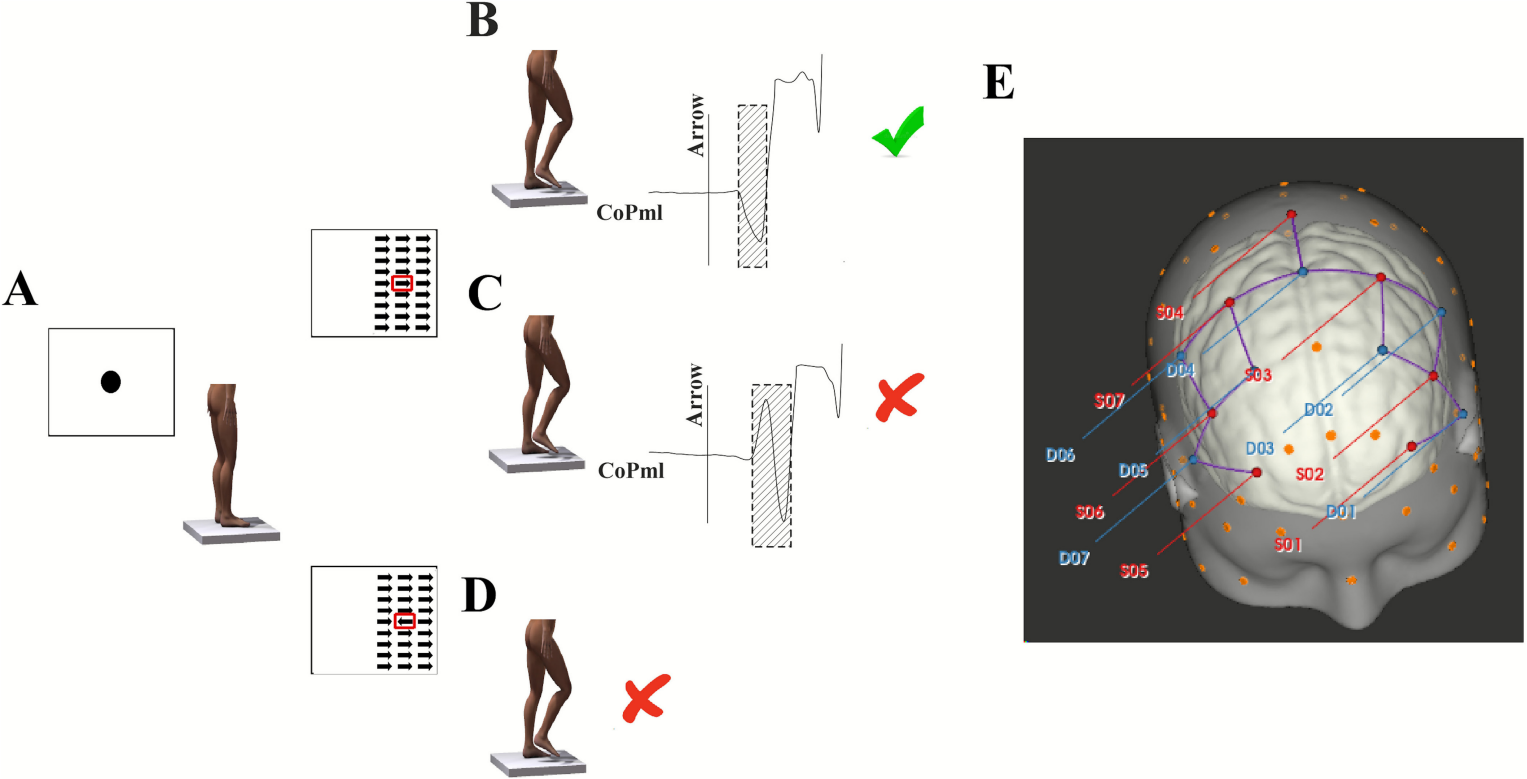
(A–D) Illustration of the task execution: (A) preparation phase, (B) correct trial (stepping with the correct leg and one APA), (C) incorrect trial (stepping with the correct leg but two APAs), (D) incorrect trial (stepping with the wrong leg). (E) Representation of the functional near‐infrared spectroscopy (fNIRS) probe layout used in the experiment, highlighting the regions of interest: supplementary motor area (SMA) and bilateral dorsolateral prefrontal cortices (DLPFC).

Participants stood on a force platform while viewing visual cues on a screen placed 3 m in front, displaying the following sequence: (1) a white screen (20 s) for mental relaxation; (2) a black circle (3 s) for task preparation (Figure 1A); and (3) a set of three columns of nine arrows (20 s) appearing on either the right or the left side of the screen. Simultaneously, a “Go!” command was played, prompting participants to quickly follow the center arrow's direction and return to the start position (Figure 1B). The imperative “GO!” cue was delivered auditorily via near‐field speakers at ~65–75 dB(A) SPL, calibrated before testing. The verbal command (“GO!”) was time‐locked to trial onset and recorded in the acquisition log for synchronization with fNIRS/force‐platform data. Two conditions were tested: congruent (arrows aligned) (Figure 1C) and incongruent (center arrow opposed) (Figure 1D). Each condition was repeated 20 times in a random order. These procedures were similar to those reported by Coelho et al. (2021). During the task, participants were instructed to look straight ahead to avoid additional changes in cerebral blood flow. We focused on trait‐level step‐initiation control and did **not** employ a FoG‐provocation protocol nor annotate FoG episodes at the trial level. APA and cortical measures were therefore analyzed as condition‐level aggregates rather than conditioned on the presence/absence of FoG events within a trial. This design isolates baseline control processes but may attenuate or amplify between‐group differences depending on whether state FoG occurred during testing.

Both biomechanical and brain function data were collected. An external trigger synchronized the motion‐capture and fNIRS systems. Biomechanical data were recorded at 200 Hz using 12 high‐speed cameras (Raptor‐4; Motion Analysis Corporation, Santa Rosa, CA, USA) and one force platform (AMTI, Watertown, MA, USA). Retroreflective markers were placed on both lateral malleoli of both legs. APA was defined by the initial lateral CoP displacement towards the leading limb, marking SI onset. APA variables analyzed were (a) APA delay: time from arrow display to APA onset, (b) APA duration: APA onset to SI, (c) APA amplitude: peak CoP displacement, and (d) task error percentage: multiple APAs or incorrect leg selection. Valid APAs required peak displacement exceeding 30% of the final mediolateral CoP displacement before stepping.

Brain function assessment used the NIRSport system (NIRx Medical Technologies, Berlin, Germany) to measure the brain's hemodynamic response, specifically the concentrations of oxyhemoglobin (oxy‐Hb) and deoxyhemoglobin (deoxy‐Hb) (Figure 1E). The setup follows the exact instructions as a previous study by Coelho et al. (2021).

Statistical channel analysis employed an autoregressive, bleached, robust regression model in NIRSLab, with general linear model (GLM) regressors for each stimulus type. Regressors included preparation (black circle), the decision‐making process (arrows indicating step direction), and the return phase (white screen). The baseline was defined as the time from the start of the step to the start of the white screen (return to the platform). A double‐gamma canonical hemodynamic response function (peak time 6 s) was used to create the regressors, adjusted for the onset and duration of each stimulus. To isolate SI‐related activity, the regressors for the black‐and‐white circle stimuli were orthogonalized with respect to the SI regressor (decision‐making process). The right inferior/middle occipital gyrus channel signal was included as a confounding regressor to account for unrelated blood flow. The SMA and bilateral DLPFC were analyzed as regions of interest (ROIs) to investigate motor preparation and cognitive conflict resolution. We evaluated hemispheric laterality by extracting time series from the right and left DLPFC and testing for hemisphere and hemisphere × group effects. As no significant differences were observed (FoG: congruent *p* = 0.961, incongruent *p* = 0.358; nFoG: congruent *p* = 0.084, incongruent *p* = 0.187), bilateral DLPFC signals were averaged to improve stability and reduce the multiple‐comparison burden. Because oxy‐HB values are more reliable and more sensitive to locomotion‐related changes in cerebral blood flow than deoxy‐HB values, only oxy‐HB concentration data were analyzed (Miyai et al. 2001).

### 2.4 Statistical Analysis

Consistent with prior evidence that SI in PD is not strongly lateralized irrespective of the more affected side (Faria et al. 2023), we prespecified a hemispheric check (right vs. left DLPFC) and, given no significant laterality, averaged bilateral signals and computed APA variables without side weighting to reduce multiplicity. Levene and Shapiro–Wilk tests assessed the data homogeneity of variances and normality. A two‐way ANOVA was performed with group (FoG and nFoG) as a between‐subject factor and condition (congruent and incongruent) as a within‐subject factor. If a significant interaction was detected, post hoc tests with a Bonferroni adjustment were applied, with a significance level set at 0.05 for all analyses. Additionally, effect size estimates were computed using eta‐squared, *η* ^*2*^. All statistical analyses were performed using Python. To probe associations between cortical responses and APAs, we conducted prespecified exploratory analyses linking APA amplitude, duration, and onset delay to fNIRS ΔHbO in SMA and DLPFC. At the subject level, we correlated each participant's mean APA metrics with mean ROI activation. These analyses are labeled exploratory and detailed in the Supporting Information.

## 3 Results

The anthropometric characteristics and clinical assessments did not differ between the two groups (Table 1).

**TABLE 1:** Means and standard deviations of the participants' characteristics and clinical assessment for each group.

	FoG (*n* = 15)	nFoG (*n* = 18)	*p*
***Participants characteristics***			
Age (years)	64.9 (7.6)	64.7 (7.9)	0.942
Height (cm)	163.8 (8.7)	169.1 (8.3)	0.087
Body mass (kg)	74.2 (14.7)	73.1 (9.7)	0.801
BMI (kg/m^2^)	27.6 (5.0)	25.6 (3.1)	0.172
Sex (male/female)	10/5	14/4	—
***Clinical assessment***			
Disease duration (years)	9.7 (2.7)	8.4 (4.1)	0.306
Hoehn & Yahr (score)	2.5 (0.5)	2.2 (0.4)	0.147
UPDRS‐III (score)	27.1 (15.5)	22.1 (11.0)	0.291
MoCA (score)	22.2 (2.7)	23.5 (3.0)	0.203

Abbreviations: MoCA, Montreal Cognitive Assessment; UPDRS‐III, motor score Unified Parkinson's disease rating.

**FIGURE 2:**
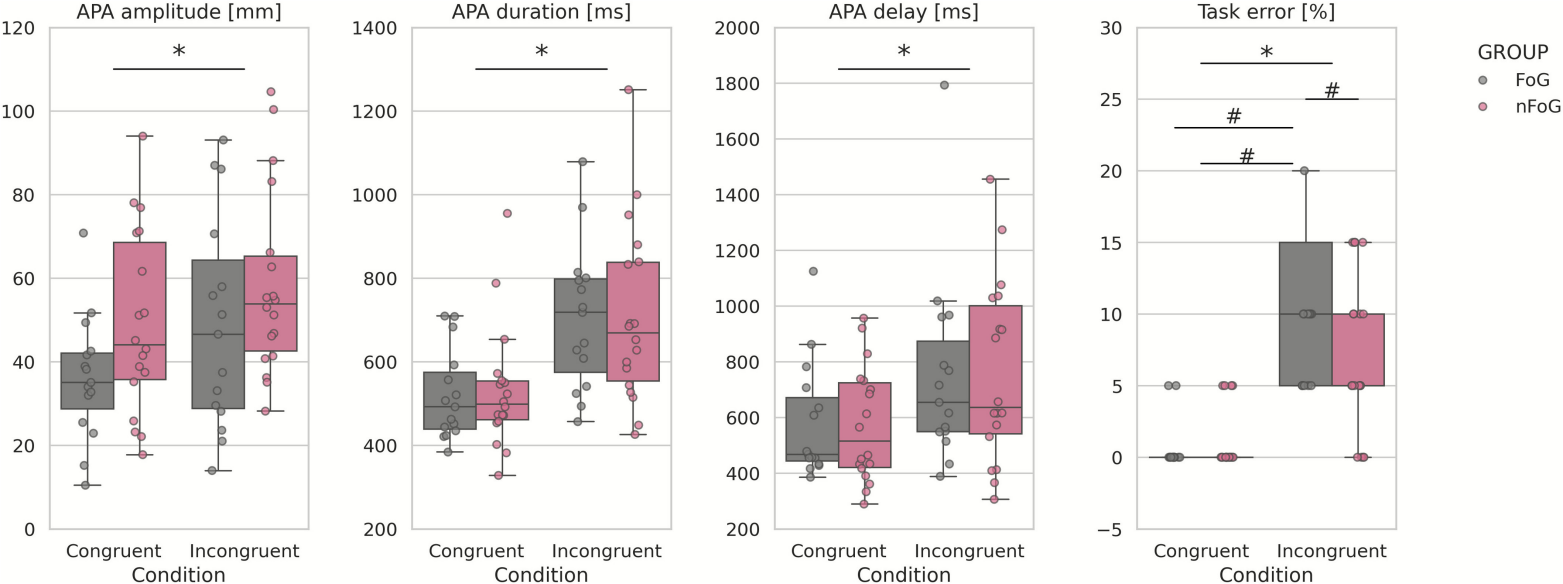
Boxplots of the APA amplitude, APA duration, APA delay, and task error (top graphs), and SMA and DLPFC activation (bottom graphs) for the FoG and nFoG groups during congruent and incongruent conditions. (*p < 0.05: statistically significant differences between conditions, +*p* < 0.05: statistically significant differences between groups, #*p* < 0.05: statistically significant interaction groups and conditions).

**FIGURE 3:**
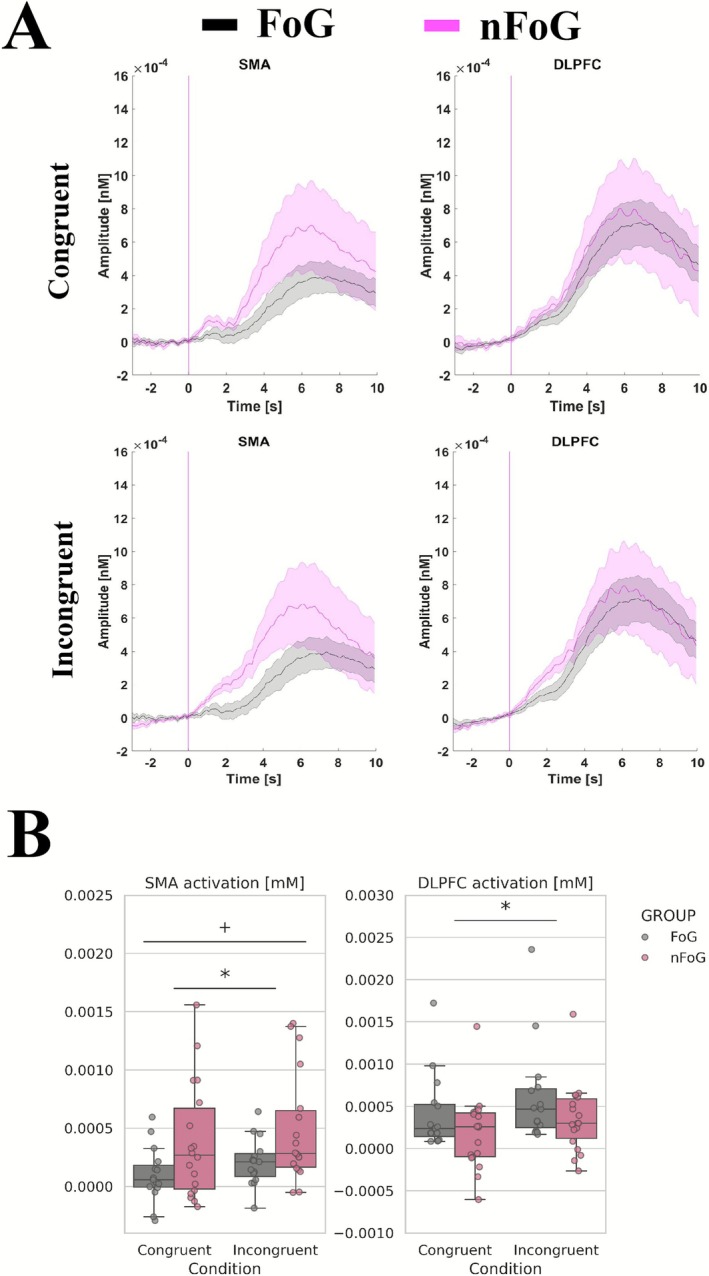
(A) Means and standard deviations of individual trials of oxy‐Hb activation of supplementary motor area (SMA) and the right and left dorsolateral prefrontal cortex (DLPFC) from SI for the FoG and nFoG for the congruent condition (top graphs) and incongruent condition (bottom graphs). (B) Boxplots of the SMA and DLPFC activation (bottom graphs) for the FoG and nFoG groups during congruent and incongruent conditions. (*p < 0.05: statistically significant differences between conditions, +*p* < 0.05: statistically significant differences between groups, #*p* < 0.05: statistically significant interaction groups and conditions).

The biomechanical assessment showed significant condition effects for the following variables: APA amplitude (*F*_1,31_ = 15.94, *p* < 0.001, *η* ^2^ = 0.34; congruent: 43.22 ± 19.97 mm, incongruent: 54.08 ± 23.95 mm), APA duration (*F*_1,31_ = 45.30, *p* < 0.001, *η* ^2^ = 0.59; congruent: 526.35 ± 129.10 ms, incongruent: 706.67 ± 194.63 ms), APA delay (*F*_1,31_ = 24.61, *p* < 0.001, *η* ^2^ = 0.44; congruent: 575.84 ± 204.59 ms, incongruent: 756.66 ± 331.14 ms), and task error (*F*_1,31_ = 27.84, *p* < 0.001, *η* ^2^ = 0.47; congruent: 0.91% ± 1.96%, incongruent: 11.21% ± 11.93%), with higher values in the incongruent condition. A group interaction difference was only found for the task error variable (*F*_1,31_ = 5.53, *p* = 0.025, *η* ^2^ = 0.34), where FoG incongruent had higher errors than FoG congruent (*p* < 0.001), nFoG congruent (*p* < 0.001), and nFoG incongruent (*p* = 0.0146) (Figure 3A). No significant group effects were observed for any variable (*p* > 0.05), indicating similar overall performance between FoG and nFoG (Figure 2).

Mean and SD of oxy‐Hb activation of the SMA and DLPFC are shown in Figure 3A. For the brain function assessment, the SMA showed a main effect of group (*F*_1,31_ = 4.48, *p* = 0.042, *η* ^2^ = 0.13), where the nFoG presented higher values than the FoG group, and a main effect of condition (*F*_1,31_ = 13.09, *p* = 0.001, *η* ^2^ = 0.30), with the incongruent condition yielding higher values than the congruent condition. However, no interaction group × condition was observed (*p* = 0.973). The oxy‐Hb for DLPFC presented a main effect of condition (*F*_1,31_ = 25.18, *p* < 0.001, *η* ^2^ = 0.45), with the incongruent condition showing greater values than the congruent condition. Furthermore, no main effect of group (*p* = 0.4515) or interaction between group and condition (*p* = 0.6717) was found, suggesting that the groups responded similarly across conditions (Figure 3B).

## 4 Discussion

This study aimed to assess executive control of inhibition by inducing cognitive‐motor conflict during foot selection for SI, comparing individuals with PD, with and without FoG. Biomechanical variables indicated impairments in APA parameters between congruent and incongruent conditions; however, contrary to our hypothesis, no significant differences were found between FoG and nFoG groups. Consistent with our hypothesis, the FoG group showed lower SMA activation than the nFoG group, highlighting potential neural deficits underlying FoG during SI in PD. An important consideration is the lack of explicit FoG triggering/measurement during testing. If FoG‐classified participants completed trials without FoG, actual group differences could be diluted; if FoG occurred in‐task, differences could be accentuated relative to no‐FoG participants. Our findings should therefore be interpreted as reflecting underlying control during SI rather than event‐locked FoG dynamics.

### 4.1 FoG vs. nFoG During SI With Cognitive‐Motor Conflict

Surprisingly, no significant differences between FoG and nFoG groups were observed for APA parameters. Individuals with FoG typically exhibited longer APA duration and smaller APA amplitude than those without FoG (Schlenstedt et al. 2018), as observed in more straightforward tasks such as the 7‐m walking test (Hou et al. 2024). However, these findings were based on straightforward motor tasks, whereas our investigation focused on an inhibition‐control task that required greater cognitive engagement. This increase in cognitive load made detecting motor differences associated with FoG more challenging.

Additionally, our participants were assessed during the “on” medication state, which may have reduced our ability to detect FoG‐specific differences, unlike previous studies that assessed participants during the “off” medication state (Hou et al. 2024; Onuma et al. 2024). An earlier study reported that FoG during SI was observed in 58% of trials in the “off” state, whereas only 5% of trials showed FoG in the “on” state (Schaafsma et al. 2003). Individuals with FoG levodopa seem to increase functional connectivity, often normalizing it to levels observed in nFoG individuals (Potvin‐Desrochers et al. 2023). Thus, the lack of findings in our study may be attributed to the potential masking effect of medication, which could have diminished the prevalence and severity of FoG during SI.

In parallel with these motor findings, we observed no significant gait asymmetry in SI, irrespective of the more affected side (Faria et al. 2023). Although PD gait is characterized by asymmetry due to uneven striatal depletion, our results support the hypothesis that FoG is mediated by bilateral network deficits rather than unilateral mechanisms (Plotnik et al. 2008). It is important to note that this functional bilaterality does not negate the clinical relevance of asymmetry. Previous studies have reported that hemispheric lateralization remains crucial for personalized interventions (Seuthe et al. 2024); for instance, asymmetric DBS targeting the most affected side has shown superior outcomes for gait and FoG compared with symmetric stimulation (Fasano et al. 2011). Thus, while the underlying circuitry benefits from lateralized treatment, the cortical strategy observed in our study suggests the brain attempts to compensate for these asymmetric deficits by engaging a broad, bilateral network.

Although FoG in PD has been associated with a decoupling between postural preparation and stepping (Jacobs, Nutt, et al. 2009), often accompanied by cognitive deficits, particularly in executive function and inhibitory control (Cohen et al. 2014), this is the first study to specifically analyze the effects of inhibition by inducing cognitive‐motor conflict at the brain's cognitive level associated with FoG during SI. Our findings revealed reduced SMA activity in individuals with FoG compared with those without FoG (nFoG), consistent with previous research identifying reduced structural and functional connectivity within the inhibitory circuit between the SMA and the STN in FoG individuals (Fling et al. 2014). However, they analyzed brain activity during resting‐state functional magnetic resonance imaging, which may not reflect real‐world scenarios encountered in the daily lives of individuals with PD. In contrast, our study focused on SI, a task that closely mirrors the motor and cognitive demands of everyday activities in this population. Given the SMA's role in generating APAs to stabilize body segments prior to movement, the decreased SMA activity observed in FoG individuals suggests an impaired ability to manage the cognitive‐motor conflict required for effective SI. Interestingly, no differences in the DLPFC were observed between FoG and nFoG groups. This contrasts with the expectation that the DLPFC, a region implicated in inhibitory control and cognitive tasks, would show reduced activity during cognitive conflict tasks in individuals with PD (Pelicioni et al. 2020). However, while participants in that study were individuals with PD, the presence of FoG was not accounted for. Thus, the lack of change in DLPFC in our study may reflect distinct neural mechanisms in individuals with PD experiencing FoG. Furthermore, as both motor and cognitive tasks were involved, the DLPFC was not solely engaged in executive control (Barbey et al. 2013). These findings suggest that the cognitive‐motor conflict in this study relies more on networks involving the SMA and other basal ganglia structures than the DLPFC in the context of FoG in individuals with PD.

Our results converge with prior evidence that modulating the SMA can ameliorate FoG in PD (Jacobs, Lou, et al. 2009; Mi et al. 2019). In a randomized controlled trial, high‐frequency rTMS over the SMA improved FoG Questionnaire scores and spatiotemporal gait measures, and a pseudorandomized parallel study reported fewer freezing episodes during SMA stimulation than during motor cortex stimulation. Mechanistically, SMA involvement in SI control and APA timing has been demonstrated with perturbational approaches, supporting the plausibility that SMA‐directed neuromodulation can influence FoG‐related gait control. Collectively, these data situate our findings within an SMA‐centric framework for SI and FoG.

### 4.2 Congruent vs. Incongruent Conditions During SI

Our findings revealed that biomechanical variables differed between the two conditions: The incongruent condition showed higher APA values and task errors than the congruent condition. Similar results have been reported in previous studies, in which cognitive‐motor conflict can increase variability in motor responses. Specifically, an unknown cue before stepping led to higher delays and multiple APAs than a known cue in older adults with PD (Cohen et al. 2017). Similarly, multiple APAs occur during incongruent conditions when younger and older adults are tested (Coelho et al. 2021). In our study, the increased APA amplitude in the incongruent condition may reflect a compensatory mechanism, suggesting that individuals with PD have greater difficulty preparing for movement. Consequently, increased task errors may indicate disrupted coordination between postural preparation and movement initiation, a pattern often observed in individuals with PD (Jacobs, Nutt, et al. 2009).

The increased APA values and task errors during cognitive‐motor conflict can be explained by heightened activity in the SMA and DLPFC regions compared to the congruent. The DLPFC, which is involved in cognitive inhibitory processes, has also shown increased hemodynamic activity during incongruent conditions in older adults (Langenecker et al. 2004). These findings suggest that inhibitory control and cognitive demand are critical factors in FoG, highlighting the complex interplay between motor and cognitive processes in gait regulation for individuals with PD. A more complex task demands greater brain processing (Van Ruitenbeek et al. 2023), leading to increased brain activity that enhances the motor program and improves motor control. Consequently, SI in PD may not only depend on motor networks but also on cognitive resources, particularly under conditions of cognitive conflict or uncertainty.

### 4.3 Limitations

Some limitations in this study should be acknowledged. We acknowledge the small sample size relative to epidemiological studies; this is common across mechanistic APA/FoG and cortical activation work and reflects feasibility constraints in deeply phenotyped protocols. Moreover, the sex imbalance in our sample (24 males/nine females) prevents the statistical analysis of sex as a biological variable. We did not include a control group of neurologically healthy individuals, which limits our ability to determine whether the observed findings are specific to FoG or simply reflect increased cognitive and motor demands during the incongruent condition. While we did not include healthy controls in this dataset—by design, to maximize power for the within‐PD contrast—recent PD‐versus‐control data in an otherwise comparable protocol are available (De Oliveira, De Oliveira, et al. 2025) to contextualize the present findings. All testing was performed in the ON‐medication state to enhance ecological validity and participant safety; while this reflects typical daily function, paired ON/OFF designs could more directly parse medication effects on APAs and cortical responses. While we avoided global Bonferroni corrections to preserve power in a mechanistic design, we acknowledge residual multiplicity risk and therefore provide effect sizes as safeguards. We did not provoke or annotate FoG episodes at the trial level. This may bias between‐group effects toward the null (no in‐task FoG) or away from it (in‐task FoG), limiting event‐specific inference. Furthermore, we averaged bilateral DLPFC signals for primary analyses, as we did not observe significant main effects by hemisphere. However, we acknowledge that PD is inherently asymmetric. Previous studies suggest that averaging functional imaging data across hemispheres may obscure side‐specific neural adaptations or compensatory mechanisms (Lizarraga et al. 2024). Thus, although our approach increased the signal‐to‐noise ratio, it may have missed lateralized cortical responses specific to the most affected side. Future work should then incorporate larger sample sizes to enable robust side‐specific comparisons. Future studies should also incorporate balanced recruitment to investigate potential sex differences, complex provocation paradigms (e.g., turning‐in‐place, narrow passage, time pressure, and dual‐task interference), and trial‐level FoG tagging (video/IMU with synchronized markers) to capture event‐related analyses of APA and cortical responses to FoG onset.

## 5 Conclusion

In summary, this study provides important insights into the neural and biomechanical underpinnings of FoG in individuals with PD during SI under cognitive‐motor conflict. Our findings revealed that individuals with PD and FoG exhibit reduced SMA activity and impaired APAs, particularly under conditions requiring inhibitory control. These results highlight the critical role of SMA activation and APA regulation in managing cognitive‐motor conflicts, suggesting that FoG is not merely a motor impairment but a complex interplay between cognitive and motor deficits. Furthermore, increased DLPFC activity under incongruent conditions underscores the cognitive demands of inhibitory control tasks and their relevance to gait disturbances. The study's potential lies in its contribution to understanding how cognitive‐motor integration impacts FoG, providing a foundation for designing targeted rehabilitation strategies that address both motor and cognitive dysfunctions. Our findings motivate targeted interventions that jointly address cognitive control and motor execution during SI. Practical options include dual‐task gait training that pairs step‐initiation practice with executive‐function drills (set‐shifting, inhibition, and working‐memory updating) and cue‐augmented training that combines visual/auditory cues with APA biofeedback to reinforce timely weight shift. Technology‐assisted approaches—such as treadmill or overground training with virtual‐reality obstacle negotiation or rhythmic auditory stimulation integrated with cognitive tasks—can simultaneously challenge planning and scaling of APAs while taxing attention. Additional candidates are action observation + motor imagery protocols focused on SI and turning/complex‐environment practice under controlled cognitive load. Where available, neuromodulation‐informed programs (e.g., SMA‐focused stimulation paired with step‐initiation training) may further enhance cognitive‐motor integration. Collectively, these strategies align with our results by targeting the interaction between cortical control and APAs to reduce FoG risk in everyday contexts.

## Author Contributions

**Claudiane Arakaki Fukuchi:** formal analysis, writing – original draft, writing – review and editing. **Luana dos Santos de Oliveira:** conceptualization, methodology, investigation. **Claudia Eunice Neves de Oliveira:** conceptualization, methodology, investigation. **Layla Cupertino:** methodology, investigation. **João Ricardo Sato:** software, writing – review and editing. **Margarete de Jesus Carvalho:** validation, writing – review and editing. **Fabio Augusto Barbieri:** validation, writing – review and editing. **Daniel Boari Coelho:** conceptualization, supervision, funding acquisition, writing – review and editing.

## Funding

This study was supported by Fundação de Amparo à Pesquisa do Estado de São Paulo (FAPESP, #2024/04859‐0), by Universidade Federal do ABC (UFABC/Brazil), by Coordenação de Aperfeiçoamento de Pessoal de Nível Superior (CAPES/Brazil) and by Conselho Nacional de Desenvolvimento Científico e Tecnológico (CNPq, #306638/2023‐1).

## Conflicts of Interest

The authors declare no conflicts of interest.

## Supporting information


**Data S1:** Supporting information.

**Data S2:** Supporting information.

## Data Availability

The data are in the Supporting Information. More information will be provided by the corresponding author.
